# The nose is not enough: Multi‐site sampling is best for MRSP detection in dogs and households

**DOI:** 10.1111/vde.13118

**Published:** 2022-08-25

**Authors:** Sian‐Marie Frosini, Ross Bond, Ruth H. King, Anette Loeffler

**Affiliations:** ^1^ Department of Clinical Sciences and Services Royal Veterinary College Hatfield Hertfordshire UK

## Abstract

**Background:**

Following recovery from meticillin‐resistant *Staphylococcus pseudintermedius* (MRSP) infection of any type, dogs may continue to carry MRSP asymptomatically on skin and mucosae, contributing to the spread of this multidrug‐resistant, veterinary hospital‐associated pathogen with zoonotic potential to others and into the environment.

**Objectives:**

This study determined which canine anatomic and household environmental sites are most sensitive for sampling to identify carriage and contamination.

**Methods and Materials:**

Fifty‐one dogs and 22 households, MRSP‐positive on at least one tested site, were sampled on 132 and 40 occasions over time, respectively. Dogs were swabbed at six sites (mouth, nose, conjunctiva, skin, prepuce/vulva, perianal area); household environments were sampled using contact plates (mannitol salt agar [MSA] and MSA + 6 mg/L oxacillin [MS+]) on five sites. MRSP was isolated after enrichment, grown on MSA/MS+ and was confirmed by PCR. Generalized estimating equations were used for calculation of sensitivity (95% confidence interval) for each site/combination.

**Results:**

Each anatomical and environmental site yielded MRSP at least once. MRSP was isolated from only a single site in 27.3% of dogs, with the buccal mucosa showing the highest sensitivity (63.8%). Multi‐site sampling of a minimum of four canine anatomical or four environmental sites, respectively, was needed to achieve >95% sensitivity.

**Conclusions and clinical relevance:**

The canine buccal mucosa should be included in MRSP sampling protocols, ideally in addition to at least three other anatomical sites. Likewise, environment sampling should be of multiple household sites in cases where it is used as a part of clinical case management.

## Abstract 2

**Background –** Following recovery from meticillin‐resistant *Staphylococcus pseudintermedius* (MRSP) infection of any type, dogs may continue to carry MRSP asymptomatically on skin and mucosae, contributing to the spread of this multidrug‐resistant, veterinary hospital‐associated pathogen with zoonotic potential to others and into the environment. **Objectives –** This study determined which canine anatomic and household environmental sites are most sensitive for sampling to identify carriage and contamination. **Conclusions and clinical relevance –** The canine buccal mucosa should be included in MRSP sampling protocols, ideally in addition to at least three other anatomical sites. Likewise, environment sampling should be of multiple household sites in cases where it is used as a part of clinical case management.

## Résumé

**Contexte:**

Après la guérison d'une infection à *Staphylococcus pseudintermedius* (MRSP) résistante à la méticilline, les chiens peuvent continuer à être porteurs asymptomatiques de MRSP sur la peau et les muqueuses, contribuant ainsi à la propagation de cet agent pathogène multirésistant, associé aux hôpitaux vétérinaires et présentant un potentiel zoonotique et dans l'environnement.

**Objectifs:**

Cette étude a déterminé quels sites anatomiques canins et environnementaux domestiques sont les plus sensibles pour l'échantillonnage afin d'identifier le portage et la contamination.

**Méthodes et matériel:**

Cinquante et un chiens et 22 habitations, positifs au MRSP sur au moins un site testé, ont été échantillonnés respectivement à 132 et 40 reprises au fil du temps. Les chiens ont été écouvillonnés à six endroits (bouche, nez, conjonctive, peau, prépuce/vulve, région périanale); les environnements domestiques ont été échantillonnés à l'aide de boîtes de contact (gélose au sel de mannitol [MSA] et MSA + 6 mg/L d'oxacilline [MS+]) sur cinq sites. Le MRSP a été isolé après enrichissement, cultivé sur MSA/MS+ et confirmé par PCR. Des équations d'estimation généralisées ont été utilisées pour le calcul de la sensibilité (intervalle de confiance à 95 %) pour chaque site/combinaison.

**Résultats:**

Chaque site anatomique et environnemental a donné au moins une fois un MRSP. Le MRSP a été isolé à partir d'un seul site chez 27,3 % des chiens, la muqueuse buccale présentant la sensibilité la plus élevée (63,8 %). Un échantillonnage multisite d'au moins quatre sites anatomiques canins ou quatre sites environnementaux, respectivement, était nécessaire pour atteindre une sensibilité > 95 %.

**Conclusions et pertinence clinique:**

La muqueuse buccale canine devrait être incluse dans les protocoles d'échantillonnage du MRSP, idéalement en plus d'au moins trois autres sites anatomiques. De même, l'échantillonnage de l'environnement doit porter sur plusieurs sites domestiques dans les cas où il est utilisé dans le cadre de la gestion des cas cliniques.

## RESUMEN

**Introducción:**

Después de recuperarse de cualquier tipo de infección por *Staphylococcus pseudintermedius* resistente a la meticilina (MRSP), los perros pueden continuar siendo portadores de MRSP de forma asintomática en la piel y las mucosas, lo que contribuye a la propagación de este patógeno, que está asociado a hospitales veterinarios y que es resistente a múltiples fármacos con potencial zoonótico a otros perros y en el medio ambiente.

**Objetivos:**

Este estudio determinó qué sitios ambientales domésticos y anatómicos caninos son más sensibles en la toma de muestras para identificar el transporte y la contaminación.

**Métodos y materiales:**

Cincuenta y un perros y 22 hogares, MRSP positivo en al menos un sitio analizado, fueron muestreados en 132 y 40 ocasiones a lo largo del tiempo, respectivamente. Se tomaron muestras de los perros en seis sitios (boca, nariz, conjuntiva, piel, prepucio/vulva, área perianal); Se tomaron muestras de ambientes domésticos usando placas de contacto (agar manitol salino [MSA] y MSA + 6 mg/L de oxacilina [MS+]) en cinco sitios. MRSP se aisló después del enriquecimiento, se cultivó en MSA/MS+ y se confirmó mediante PCR. Se usaron ecuaciones de estimación generalizadas para el cálculo de la sensibilidad (intervalo de confianza del 95 %) para cada sitio/combinación.

**Resultados:**

Cada sitio anatómico y ambiental arrojó MRSP al menos una vez. MRSP se aisló de un solo sitio en el 27,3 % de los perros, y la mucosa bucal mostró la mayor sensibilidad (63,8 %). Se necesitó un muestreo multilocalización de un mínimo de cuatro sitios anatómicos caninos o cuatro ambientales, respectivamente, para lograr una sensibilidad >95 %.

**Conclusiones y relevancia clínica:**

la mucosa bucal canina debe incluirse en los protocolos de muestreo de MRSP, idealmente además de al menos otros tres sitios anatómicos. Del mismo modo, el muestreo ambiental debe ser de múltiples localizaciones domésticas en los casos en que se utilice como parte del manejo de casos clínicos.

## Zusammenfassung

**Hintergrund:**

Nach der Erholung von einer Infektion mit einem Methicillin‐resistenten *Staphylococcus pseudintermedius* (MRSP) eines jeden Typs können Hunde weiterhin asymptomatische Träger von MRSP auf der Haut und auf den Schleimhäuten sein, was zu einer Weiterverbreitung dieses multi‐resistenten Keims, welcher mit Tierarztpraxen in Zusammenhang steht, zoonotisches Potential für andere und für die Umgebung aufweist, beiträgt.

**Ziele:**

Diese Studie identifizierte die anatomischen Körperstellen der Hunde sowie die Umweltlokalisationen des Haushalts, die am sensibelsten sind, um Träger und Kontamination zu identifizieren.

**Methoden und Materialien:**

Von einundfünfzig Hunden und 22 Haushalten, die an mindestens einer Stelle MRSP‐positiv getestet waren, wurde 132 bzw 40‐mal im Verlauf der Zeit Proben genommen. Den Hunden wurde mittels Tupfer an sechs Stellen Proben entnommen (Mund, Nase, Bindehaut, Haut, Präputium/Vulva, Perianalgegend); in der Haushaltsumgebung wurden mittels Kontaktplatten (Mannitolsalzagar [MSA] und MSA + 6 mg/L Oxacillin [MS+]) an fünf Stellen Proben entnommen. Ein MRSP konnte nach einer Anreicherung isoliert werden, wuchs auf MSA/MS+ und wurde mittels PCR bestätigt. Generalisierte Schätzgleichungen wurden zur Kalkulierung der Sensibilität (95% Konfidenzintervall) für jede Stelle/Kombination angewendet.

**Ergebnisse:**

Jede anatomische und Umweltstelle lieferte mindestens einmal einen MRSP. MRSP wurde nur von einer einzigen Stelle bei 27,3% der Hunde isoliert, wobei die Backenschleimhaut die höchste Sensibilität (63,8%) aufwies. Eine multiple Probenahme von mindestens vier anatomischen bzw vier Umweltstellen war nötig, um eine >95%ige Sensibilität zu erreichen.

**Schlussfolgerungen und klinische Bedeutung:**

Die Backenschleimhaut des Hundes sollte bei MRSP Probenahme Protokollen inkludiert werden, idealerweise zusätzlich zu mindestens drei anderen anatomischen Körperstellen. Ebenso sollte die Probenahme aus der Umwelt an multiplen Stellen im Haushalt erfolgen, wenn es sich um Fälle handelt, wo es als Teil des klinischen Managements eingesetzt wird.

## 要約

**背景:**

メチシリン耐性ブドウ球菌 (MRSP) 感染から回復後も、犬は皮膚や粘膜に無症候性にMRSP を保持し続け、この多剤耐性動物病院関連病原体を他人や環境へ拡散させる可能性がある。

**目的:**

本研究の目的は、保菌や汚染を特定するためのサンプリングに最も感度が高い犬の解剖学的部位および家庭環境を特定することであった。

**材料と方法:**

少なくとも 1 つの検査部位で MRSP 陽性を示した 51 頭の犬および22 世帯を、それぞれ 132 回および40 回にわたりサンプリングした。犬は 6 部位 (口、鼻、結膜、皮膚、包皮/外陰部、肛門周囲) を綿棒で拭き取り、家庭環境は、5 箇所でコンタクトプレート (マンニトール塩寒天 [MSA] および MSA + 6 mg/L オキサシリン [MS+]) を使用してサンプリングされた。MRSPは濃縮後に分離し、MSA/MS+で増殖させ、PCRで確認した。一般化推定方程式を用いて、各部位/組み合わせごとに感度 (95% 信頼区間)を算出した。

**結果:**

各解剖学的部位および環境箇所から少なくとも 1 回 MRSP が検出された。 MRSP は 27.3% の犬で単一部位から分離され、頬粘膜が最も高い感度 (63.8%) を示した。 95% 以上の感度を達成するには、それぞれ犬から最低 4 解剖学的部位または 4環境箇所のマルチサイト サンプリングが必要であった。

**結論と臨床的関連性:**

犬頬粘膜はMRSPサンプリングプロトコルに含めるべきであり、理想的には少なくとも他の3つの解剖学的部位に加えて含めるべきである。同様に、環境サンプリングは、臨床的な症例管理の一部として使用する場合には、複数の家庭用部位を対象とすべきである。

## 摘要

**背景:**

从任何类型的耐甲氧西林假中间葡萄球菌 (MRSP) 感染中恢复后，犬的皮肤和粘膜可能继续无症状地携带MRSP，导致这种具有人畜共患病潜力的多重耐药、兽医医院相关病原体传播给其他人和进入环境。

**目的:**

本研究确定了哪些犬解剖和家庭环境部位的携带和污染，采样识别最敏感。

**方法和材料:**

51只犬和22户家庭，至少一个检测点 MRSP 阳性，随时间推移分别采样132次和40次。在6个部位(口、鼻、结膜、皮肤、包皮/外阴、肛周)擦拭犬；在5个部位使用接触平板(甘露醇盐琼脂 [MSA] 和MSA + 6 mg/L苯唑西林 [MS +])对家庭环境进行采样。富集后分离MRSP，在MSA/MS + 上生长并经 PCR 证实。使用广义估计方程计算每个研究中心/组合的敏感性(95%置信区间)。

**结果:**

每个解剖和环境部位至少产生一次MRSP。27.3%的犬仅从单个部位分离出MRSP，其中颊黏膜表现出最高的敏感性 (63.8%)。分别需要对至少4个犬解剖部位或4个环境部位进行多部位采样，敏感性达到 > 95%。

**结论和临床相关性:**

除至少3个其他解剖部位外，理想情况下，MRSP采样方案中应包括犬颊粘膜。同样，应在多个家庭位点进行环境采样，选择临床病例管理环境。

## Resumo

**Contexto:**

Após a recuperação de qualquer tipo de infecção por *Staphylococcus pseudintermedius* resistente à meticilina (MRSP), os cães podem continuar a ser portadores MRSP de forma assintomática na pele e mucosas, contribuindo para a disseminação deste patógeno multirresistente, associado ao hospital veterinário com potencial zoonótico para outros e no ambiente.

**Objetivos:**

Este estudo determinou quais locais anatômicos caninos e domiciliares são mais sensíveis para amostragem para identificação de portadores e contaminação.

**Métodos e Materiais:**

Cinquenta e um cães e 22 domicílios, MRSP‐positivos em pelo menos um local testado, foram amostrados em 132 e 40 ocasiões ao longo do tempo, respectivamente. Coletou‐se as amostras utilizando‐se swabs em seis locais (boca, nariz, conjuntiva, pele, prepúcio/vulva, região perianal); os ambientes domiciliares foram amostrados utilizando placas de contato (ágar sal manitol [MSA] e MSA + 6 mg/L de oxacilina [MS+]) em cinco locais. MRSP foi isolado após enriquecimento, cultivado em MSA/MS+ e confirmado por PCR. Equações de estimativa generalizada foram usadas para cálculo de sensibilidade (intervalo de confiança de 95%) para cada local/combinação.

**Resultados:**

Cada sítio anatômico e ambiental apresentou MRSP pelo menos uma vez. MRSP foi isolado de um único sítio em 27,3% dos cães, com a mucosa oral apresentando a maior sensibilidade (63,8%). A amostragem em vários locais de um mínimo de quatro locais anatômicos caninos ou quatro locais ambientais, respectivamente, foi necessária para atingir > 95% de sensibilidade.

**Conclusões e relevância clínica:**

A mucosa oral canina deve ser incluída nos protocolos de amostragem de MRSP, idealmente além de pelo menos três outros sítios anatômicos. Da mesma forma, a amostragem do ambiente deve ser de vários locais domiciliares nos casos em que é usada como parte do gerenciamento de casos clínicos.

**Abbreviations**
CI: confidence interval
GEE: generalised estimating equations
MDR: multidrug‐resistant
MRSA: Meticillin‐resistant *Staphylococcus aureus*
MRSP: Meticillin‐resistant *Staphylococcus pseudintermedius*
MS+: Mannitol salt agar supplemented with 6 mg/L oxacillin
MSA: Mannitol salt agar
MSSP: Meticillin‐susceptible *Staphylococcus pseudintermedius*
PCR: Polymerase chain reaction
RVC: Royal Veterinary College
TSB: Tryptone soy broth

## INTRODUCTION

Over the past 15 years, meticillin‐resistant *Staphylococcus pseudintermedius* (MRSP) has become the major multidrug‐resistant (MDR) bacterial pathogen in canine skin and soft‐tissue infections.^1^ Like other staphylococci, MRSP adheres to squames and hair after infection has resolved. Such nonclinical MRSP carriage poses a risk to the host if infection recurs, as seen in human *Staphylococcus aureus* infections,^2^ and contributes to transmission to in‐contact humans and dogs. Implementation of infection control measures preventing nosocomial spread of MRSP requires accurate identification of carriers, of especial importance in settings with low MRSP prevalence, such as the UK.

Screening for MRSA carriage in humans before hospital admission or elective procedures is routine worldwide, within the boundaries of funding and practicability. Systematic review indicates that the most sensitive single human swabbing site is the nose, detecting 68% of carriers (34%–91%),^3^ and this can be increased to almost 95% sensitivity by combining throat, groin and nose.^3^

Conversely, nasal swabbing in dogs is reported to be less sensitive than in humans to identify *S. pseudintermedius* carriers (16%–64%), with the perineum and mouth cited as predominant carriage sites.^4^, ^5^ In a single study, analysis of MRSP recovery from 73 sample sets from 27 dogs covering four sites (nose, mouth, perineum, pharynx) identified similar predilection sites for MRSP to meticillin‐susceptible *S. pseudintermedius* (MSSP), with perineal (63%) and corner of the mouth (58%) sampling being most sensitive.^6^

The household environment has been highlighted for potential contamination by MRSP‐carrier dogs, providing a source for future reinfection. Previous studies identified MRSP most commonly in dog‐accessible areas, such as feeding and sleeping places.^7^ Further investigation is needed to inform on the utility of household sampling when managing recurrent MRSP infections that do not relate to MRSP carriage on mucosal sites.

Previous sampling studies for MRSP carriage in dogs have focussed on the nose and perineum, extrapolated from nasal swabbing for human MRSA carriage, and ease of sampling the perineum. This study aimed to determine the sensitivity of swab sampling six anatomical sites for detecting canine MRSP carriage, and sensitivity of contact plate sampling of five household environment sites for environmental contamination.

## MATERIALS AND METHODS

### Ethics

This study was approved by the Royal Veterinary College's (RVC) Clinical Research Ethical Review Board (URN 2012 1166); owners gave written consent at enrolment.

### Study population and design

Dogs had been recruited as part of a study investigating the efficacy of topical antimicrobial therapy for eradicating MRSP carriage after a previous episode of MRSP infection had resolved. They had been enrolled either at the Queen Mother Hospital for Animals (RVC) or via referring veterinary surgeons between May 2016 and December 2019.

### Sampling for dog carriage of and household contamination with MRSP

Six anatomical sites were sampled from each dog: buccal mucosa inside the upper lip, outside the teeth; nose including nasal planum and one or both nostrils; conjunctival mucosa uni‐ or bilaterally; axilla or groin skin; preputial or vulval mucosa; and perianal area where nonhaired meets haired skin. Dry sterile cotton swabs (charcoal transport swabs, SLS) were rolled over each site for 3–5 s.

For household contamination, five different sites in the room most frequently occupied by the dog were sampled: floor; frequently cleaned hard hand‐touch surface (e.g. kitchen work surface); infrequently cleaned inaccessible surface (e.g. top of cupboard); dog bed; and dog bowl. Owners were instructed to place paired mannitol salt agar (MSA; CM0085, ThermoScientific) and MSA supplemented with 6 mg/L oxacillin (MS+) 55 mm contact plates (ThermoScientific) on each site for 5 s.

‘Sampling event’ was used to describe each time at which a full set of samples (six anatomic or five environmental) was taken.

### Isolation and confirmation of MRSP

Swabs and plates were posted to the RVC for microbiological analyses and processed immediately on receipt. Swabs were incubated individually in tryptone soy broth (TSB; CM0129, ThermoScientific) supplemented with 10% sodium chloride (Sigma‐Aldrich Ltd) at 37°C for 48 h. Broth aliquots were subcultured onto MSA and MS+ and incubated at 37°C for 24–48 h. Contact plates were incubated at the laboratory at 37°C for 48 h. If no growth was observed after 48 h, the sample was discarded.

Each distinct, presumed staphylococcal, colony from MSA and MS+ was subcultured onto blood agar base (CM0271; Thermo Scientific) containing 5% sheep blood (TCS BioScience) after morphological assessment based on size (small–medium), shape (round with regular edges) and colour (white to cream) of colonies. Growth was further characterized phenotypically for clumping factor ability using dog plasma, DNase production and by Voges‐Proskauer testing.^8^ Suspected MRSP were confirmed by PCR, demonstrating the presence of species‐specific thermonuclease, *nuc*, and *mecA*.^8^

### Statistical methods

Results of sampling events were analysed for dogs/environments testing positive for MRSP at a minimum of one site. Generalized estimating equations (GEE), accounting for repeated measures of some dogs or environments, were used to determine sensitivity (95% confidence interval [CI]) of each site, and combinations of sites, in detecting MRSP (Spss Statistics v26, IBM). GEE used dog or environment as the subject variable, with an exchangeable working correlation matrix in a binary logistic regression model. Comparison of sensitivity of single sites used the same GEE with site included as a linear predictor, significance *p* ≤ 0.05 (Spss Statistics v26).

## RESULTS

One‐hundred thirty‐two sampling events from 51 dogs (≤12 repeated samples from the same dog) and 40 sampling events from 22 household environments (up to four repeated samples from a single environment) were available for analyses (for sensivities of all sites and combinations, see Table S1).

Each of the six anatomical sites and five environmental sites yielded MRSP at least once, although the pattern of MRSP recovery varied between individuals and within repeated samples from the same individuals.

Meticillin‐resistant *Staphylococcus pseudintermedius* was isolated from only a single canine carriage site in 36 of 132 (27.3%) sampling events (24 of 51 dogs). In 16 of 132 (12.1%) sampling events, all six swabs yielded MRSP (13 of 51 dogs). The buccal mucosa most frequently yielded MRSP (Table 1) and was a significantly more sensitive sampling site than either axilla/groin skin (*p* < 0.0005) or prepuce/vulva (*p* = 0.008). Nose, conjunctiva and prepuce/vulva were significantly more likely to yield MRSP than axilla/groin skin (*p* = 0.002, *p* = 0.016 and *p* = 0.011, respectively). No other significant differences were seen. To achieve sensitivity ≥95%, at least four sites needed to be swabbed, always including both buccal mucosa and nose (Table 2).

**TABLE 1:** Sensitivity of sampling individual canine anatomical and household environmental sites for detecting MRSP carriage or contamination in 51 dogs (132 sampling events) and 22 households (40 sampling events), respectively

Site	Number of positive sampling events in this site/Total number of MRSP‐positive dogs or households^a^	Sensitivity (%) (95% CI)
Dog		
Buccal	83/132	64 (54–72)
Nasal	77/132	60 (37–58)
Conjunctival	67/132	48 (37–58)
Axilla/ groin skin	48/132	36 (28–45)
Prepuce/ vulva	63/132	48 (39–56)
Perianal	62/132	44 (33–56)
Environment		
Dog's bed	24/40	55 (37–72)
Dog's bowl	12/40	30 (18–46)
Floor	15/40	36 (22–53)
Frequently cleaned	2/40	5 (1–18)
Infrequently cleaned	8/40	21 (10–37)

Abbreviation: CI, confidence interval; MRSP, meticillin‐resistant *Staphylococcus pseudintermedius*.

^a^ Every sampling event recovered MRSP from at least one site, and thus, this column represents traditional sensitivity of (number true positive)/(total number positive).

**TABLE 2:** Combinations of sampling sites (canine anatomical and household environmental) to achieve ≥95% sensitivity for detecting MRSP carriage or contamination in 51 dogs (132 sampling events) and 22 households (40 sampling events), respectively

Combination of sites	Number of positive sampling events in this combination of sites/Total number of MRSP‐positive dogs or households^a^	Sensitivity (%) (95% CI)
Dog		
4 sites sampled		
B N C S	128/132	97 (93–99)
B N C PrV	126/132	96 (87–99)
B N S PrV	125/132	95 (91–98)
B N P PrV	124/132	95 (86–98)
5 sites sampled		
B N C S PrV	131/132	99 (95–100)
B N C S P	129/132	98 (94–99)
B N S PrV P	129/132	97 (92–99)
B N C PrV P	127/132	97 (87–99)
Environment		
4 sites sampled		
Dog's bed, dog's bowl, floor, infrequently cleaned	39/40	98 (85–100)

Abbreviations: B, buccal; C, conjunctival; CI, confidence interval; MRSP, meticillin‐resistant *Staphylococcus pseudintermedius*; N, nasal; S, axilla/groin skin; P, perianal; PrV, prepuce/vulva.

^a^ Every sampling event recovered MRSP from at least one site, and thus, this column represents traditional sensitivity of (number true positive)/(total number positive).

In the household environment, MRSP was identified from only a single site in 23 of 40 (57.5%) of sampling events (17 of 22 households), and none yielded MRSP from all sites at the same time. MRSP was most frequently recovered from the dog's bed (Table 1). The bed was more sensitive than the bowl (*p* = 0.037) and infrequently cleaned area (*p* = 0.003), and the bed, bowl and floor were more sensitive than the frequently cleaned site (*p* < 0.0005, *p* = 0.003 and *p* = 0.001, respectively). A sensitivity ≥95% was achieved only by combining at least four sites: the dog's bed, bowl, floor and infrequently cleaned site (Table 2).

## DISCUSSION

These results confirm that the buccal mucosa is comparable to the human nose^3^ as the most sensitive sampling site for investigating canine MRSP carriage. This is encouraging as using a swab from the inside of the lip will be better tolerated by most dogs and be safer for the sampling person than inserting a swab into nostrils. However, desirable sensitivities of ≥95% were achieved only by combining results from at least four anatomical sites. This increased sensitivity may be a consequence of either different niches being accessed or larger total surface areas being sampled. This corroborates earlier MRSP screening recommendations^7^ and mirrors findings from human medicine regarding MRSA.^3^

In general, recovery of MRSP was comparable to that reported previously for MSSP (60% vs. 16%–64% nasal; 44% vs. 28%–72% perineum).^4^ Combining buccal and perineal swabbing resulted in a much lower sensitivity than that reported previously (76% vs. 90%)^9^; further screening of MRSP versus MSSP would be needed to confirm whether this is a true difference in carriage site preference. Variability in the data across study groups indicates the importance of multi‐site swabbing to detect all carriers. If the nose cannot be sampled, our data indicate that 9% of canine MRSP carriers would be missed despite sampling all other sites, which is comparable to the reported figure of 5%–7% of humans who had MRSA recovered only from nasal swabs.^10^

Although not investigated in this study, pooling samples from different sites for processing where only a binary outcome report of MRSP carriage is needed, may be considered to reduce cost. Comparable results (93%–97% agreement) to individual culture have been reported for pooled MRSA swab processing,^11^ and further confirmation of this approach for MRSP is warranted.

For environmental sampling, best sites remain uncertain. Although all sites yielded MRSP at least once, the dog bed showed a moderate sensitivity of 55%, while yield from other sites was comparatively low. ‘Hand‐touch areas’ in human medicine have been identified as preferred sampling sites owing to their importance in MRSA‐transmission.^12^ However, extrapolation to ‘nose‐touch sites’ in a dog‐MRSP setting cannot be supported by these findings. Household sampling may be desirable for research into the role of the environment in pathogen dissemination, or for control of recurrent MRSP infections that do not appear to relate to carriage of the isolate and may be related to environmental contamination. Thus, use of environmental screening may be a rare consideration in management of clinical cases (e.g. a human at high risk of MRSP infection within the household). Sensitivities identified for environmental sites in this study indicate that multi‐site sampling is needed.

In conclusion, buccal mucosa, nose and at least two additional sites should be swabbed to maximize detection of MRSP carrier dogs. Overall, these findings should be incorporated into veterinary infection control protocols to minimize the impact of canine MRSP carriers within the veterinary practice through accurate detection.

## AUTHOR CONTRIBUTIONS

**Sian‐Marie Frosini:** Conceptualization; formal analysis; investigation; methodology; writing – original draft; writing – review and editing. **Ross Bond:** Conceptualization; funding acquisition; investigation; methodology; writing – review and editing. **Ruth H King:** Investigation; writing – review and editing. **Anette Loeffler:** Conceptualization; funding acquisition; investigation; methodology; writing – original draft; writing – review and editing.

## FUNDING INFORMATION

SMF and this study were funded by a Biotechnology and Biological Sciences Research Council (Swindon, UK; BBSRC) industrial CASE scholarship in partnership with Dechra Veterinary Products Limited (Shropshire, UK; grant no. BB/K011952/1).

## CONFLICT OF INTEREST

Design of the study; collection, analysis, and interpretation of data; and writing the manuscript, were undertaken independently of the funding body.

## Supporting information


Table S1
