# Supplementary material for: The nose is not enough: Multi‐site sampling is best for MRSP detection in dogs and households
Source: Vet Dermatol. 2022 Aug 25;33(6):576–80. doi: 10.1111/vde.13118 (PMC9804885; doi:10.1111/vde.13118)
Supplement: Supplementary file 1 — Table S1 [file VDE-33-576-s001.docx]

### Supplementary table 1: Sensitivity of different sampling sites and combinations of sites for detecting MRSP carriage in dogs (51 dogs on 132 sampling occasions) and MRSP contamination in their household environments (22 households on 40 sampling occasions).

|  | Dog  (n = 132 sampling events) | | Environment  (n = 40 sampling events) | |
| --- | --- | --- | --- | --- |
|  | Site / Combination of sites | Sensitivity (%)  (95% CI) | Site / Combination of sites | Sensitivity (%)  (95% CI) |
| Single Site | Buccal | 64 (54-72) | Dog’s bed | 55 (37-72) |
|  | Nasal | 60 (37-58) | Dog’s bowl | 30 (18-46) |
|  | Conjunctival | 48 (37-58) | Floor | 36 (22-53) |
|  | Axilla / groin skin | 36 (28-45) | Frequently cleaned | 5 (1-18) |
|  | Prepuce / Vulva | 48 (39-56) | Infrequently cleaned | 21 (10-37) |
|  | Perianal | 44 (33-56) |  |  |
| Two Sites | B N | 85 (76-90) | Bed, Bowl | 72 (53-85) |
|  | B C | 77 (68-85) | Bed, Floor | 74 (56-86) |
|  | B S | 74 (67-81) | Bed, Fr Cl | 58 (41-74) |
|  | B PrV | 75 (67-82) | Bed, Inf Cl | 69 (49-84) |
|  | B P | 76 (66-84) | Bowl, Floor | 60 (42-76) |
|  | N C | 76 (66-84) | Bowl, Fr Cl | 32 (20-48) |
|  | N S | 69 (62-76) | Bowl, Inf Cl | 49 (35-63) |
|  | N PrV | 75 (67-82) | Floor, Fr Cl | 42 (26-59) |
|  | N P | 74 (64-82) | Floor, Inf Cl | 53 (39-68) |
|  | C S | 60 (49-70) | Fr Cl, Inf Cl | 26 (14-42) |
|  | C PrV | 61 (50-71) |  |  |
|  | C P | 61 (49-72) |  |  |
|  | S PrV | 58 (48-67) |  |  |
|  | S P | 57 (47-68) |  |  |
|  | P PrV | 57 (46-67) |  |  |
| Three Sites | B N C | 92 (83-96) | Bed, Bowl, Floor | 90 (77-96) |
|  | B N S | 86 (77-92) | Bed, Bowl, Fr Cl | 77 (61-88) |
|  | B N PrV | 92 (84-96) | Bed, Bowl, Inf Cl | 85 (65-94) |
|  | B N P | 89 (81-94) | Bed, Floor, Fr Cl | 76 (58-88) |
|  | B C S | 84 (76-90) | Bed, Floor, Inf Cl | 82 (64-92) |
|  | B C PrV | 82 (72-89) | Bed, Fr Cl, Inf, Cl | 72 (53-85) |
|  | B C P | 82 (72-89) | Bowl, Floor, Fr Cl | 63 (44-79) |
|  | B S PrV | 81 (73-87) | Bowl, Floor, Inf Cl | 75 (60-86) |
|  | B S P | 82 (74-88) | Bowl, Fr Cl, Inf Cl | 50 (36-63) |
|  | B PrV P | 82 (73-89) | Floor, Fr Cl, Inf Cl | 58 (42-72) |
|  | N C S | 83 (77-88) |  |  |
|  | N C PrV | 83 (75-88) |  |  |
|  | N C P | 80 (69-88) |  |  |
|  | N S PrV | 80 (73-86) |  |  |
|  | N S P | 81 (73-87) |  |  |
|  | N PrV P | 82 (74-88) |  |  |
|  | C S PrV | 67 (55-76) |  |  |
|  | C S P | 69 (57-78) |  |  |
|  | C P PrV | 68 (56-78) |  |  |
|  | S PrV P | 66 (55-76) |  |  |
| Four Sites | B N C S | 97 (93-99) | Bed, Bowl, Floor, Fr Cl | 93 (80-98) |
|  | B N C PrV | 96 (87-99) | Bed, Bowl, Floor, Inf Cl | 98 (85-100) |
|  | B N C P | 92 (84-97) | Bed, Bowl, Fr Cl, Inf Cl | 89 (74-96) |
|  | B N S PrV | 95 (91-98) | Bed, Floor, Fr Cl, Inf Cl | 85 (65-94) |
|  | B N S P | 92 (91-92) | Bowl, Floor, Fr Cl, Inf Cl | 78 (62-88) |
|  | B N P PrV | 95 (86-98) |  | |
|  | B C P PrV | 86 (76-92) |  |  |
|  | B C S PrV | 86 (78-92) |  |  |
|  | B C S P | 88 (79-93) |  |  |
|  | B S P PrV | 87 (79-92) |  |  |
|  | N C S PrV | 87 (81-92) |  |  |
|  | N C S P | 87 (79-92) |  |  |
|  | N S PrV P | 87 (80-92) |  |  |
|  | N C PrV P | 85 (77-91) |  |  |
|  | C S PrV P | 73 (62-82) |  |  |
| Five Sites | B N C S PrV | 99 (95-100) |  |  |
|  | B N C S P | 98 (94-99) |  |  |
|  | B N C PrV P | 97 (87-99) |  |  |
|  | B C S PrV P | 91 (82-96) |  |  |
|  | N C S PrV P | 90 (84-95) |  |  |

B: buccal; N: nasal; C: conjunctival; S: axilla/groin skin; PrV: prepuce/vulva; P: perianal

Fr Cl: Frequently cleaned; Inf Cl: Infrequently cleaned
